# Vasoprotection by Dietary Supplements and Exercise: Role of TNF**α** Signaling

**DOI:** 10.1155/2012/972679

**Published:** 2011-11-01

**Authors:** Hanrui Zhang, Cuihua Zhang

**Affiliations:** Division of Cardiovascular Medicine, Departments of Internal Medicine, Medical Pharmacology and Physiology, and Nutritional Sciences, Dalton Cardiovascular Research Center, University of Missouri, Columbia, MO 65211, USA

## Abstract

Vascular dysfunction contributes to the pathogenesis of various cardiovascular diseases. Dietary supplements, including fish oil, dietary fibers, and various natural products, and exercise training exert vasoprotective effects. However, the mechanisms underlying the vasoprotective benefits of dietary supplements and physical activity demand extensive investigation. Accumulating evidence suggests that inflammatory cytokine tumor necrosis factor-alpha (TNF**α**) plays a pivotal role in the dysregulation of macrovascular and microvascular function. TNF**α** induces vascular inflammation, monocyte adhesion to endothelial cells, vascular oxidative stress, apoptosis, and atherogenic response and participates in the regulation of thrombosis and coagulation through multiple signaling pathways involving NF**κ**B, Sp1, activator protein 1, JNK, p38, STAT3, and so forth. Dietary supplements and exercise training decrease TNF**α** production and ameliorate TNF**α**-mediated pathological changes in vasculature. Thus, the inhibitory effects of dietary supplements and physical exercise on TNF**α** production and TNF**α** signaling may contribute to their vasoprotective properties.

## 1. Introduction

**Figure 1:**
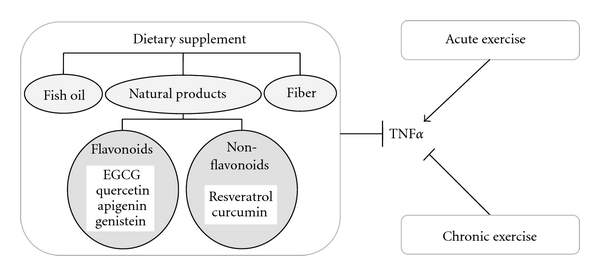
Dietary supplements (fish oil, dietary fiber, and various flavonoid and nonflavonoid natural products) exert vasoprotective benefits by inhibiting TNF*α* production and its downstream signaling pathways. There are dichotomies in the effects of exercise training on TNF*α* production. Whereas acute exercise has been reported to increase the production of proinflammatory cytokines, chronic exercise reduces TNF*α* levels.

**Figure 2:**
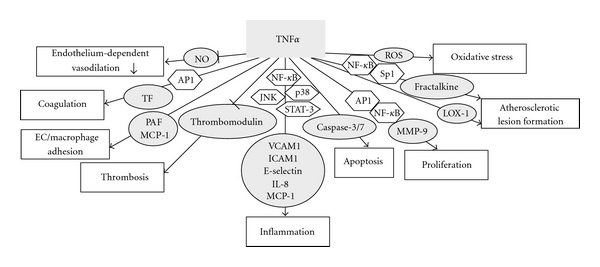
Multiple mechanisms are involved in the vasoprotective effects of dietary supplements and exercise by inhibiting TNF*α* signaling.

Endothelial dysfunction is an early indicator of cardiovascular diseases, including that seen in type 2 diabetes [1]. Accumulating evidence suggests that mediators of inflammation may be pathogenic by inducing vascular dysfunction [2]. Among the various inflammatory factors, tumor necrosis factor-alpha (TNF*α*) plays a pivotal role in the regulation of vascular function in various pathological status [3]. TNF*α* belongs to the TNF superfamily and is produced by many cell types, including macrophages, lymphocytes, and fibroblasts, and so forth [4–6]. TNF*α* can either exist in a membrane-bound form or be secreted as a soluble cytokine that is able to diffuse from the sites of its initial production [7]. It can bind to, and thus functions through its receptors, TNF receptor type 1 (TNFR1) and TNF receptor type 2 (TNFR2) [8]. Dietary supplements and exercise are emerging as effective adjunctive therapies targeting endothelial dysfunction and vascular wall inflammation [3, 9, 10]. This review summarized the vasoprotective effects of dietary supplements and exercises by inhibiting TNF*α* production and TNF*α*-induced signaling (Figures 1 and 2). 

## 2. Dietary Supplements

During the past few decades, there has been renewed interest in dietary components that might favorably reduce risk of vascular dysfunction. Fish oil, dietary fiber, and various natural products have sparked intense interest in epidemiological studies. Extensive exploration of the underlying mechanisms of their healthy benefits became very important to develop new adjunctive therapies from those dietary supplements in order to delay or prevent the development of vascular disorders. 

### 2.1. Fish Oil

Fish and other marine life serve as rich sources of a class of polyunsaturated fatty acids [11]. They are named as omega-3 or n-3 fatty acids because the first of the several double bonds occur three carbon atoms away from the terminal end of the carbon chain [11]. The three n-3 polyunsaturated fatty acids (n-3 PUFAs) include alpha linolenic acid (LNA), eicosapentenoic acid (EPA), and docosahexenoic acid (DHA) [12]. The role of fish oil lipids in regulating TNF*α* expression and TNF*α*-induced inflammation in vascular cells has been extensively studied in the past decades. Dietary supplementation with n-3 PUFA improved endothelial function and reduced circulating level of TNF*α* in offspring of patients with type 2 diabetes [13]. Omega-3 fatty acid markedly suppressed the production of TNF*α* by monocytes in response to endotoxin. In addition, it also significantly inhibited both monocyte-endothelium adhesion and transendothelial monocyte migration [14]. One of the mechanisms by which fish oil alters the ability of lymphocytes to bind to endothelial cells may be its effects on inhibiting adhesion molecule expression induced by proinflammatory cytokines such as TNF*α* [15, 16]. Moreover, dietary fish oil resulted in a 50% reduction in concanavalin A (Con A), which enhanced lymphocyte adhesion to TNF*α*-stimulated endothelial cells [17]. EPA and DHA inhibited TNF*α*-induced monocyte adhesion to activated endothelial cells in vitro by affecting endothelial platelet-activating factor (PAF) generation [18]. Dietary fish oil supplementation inhibited TNF*α* production by human peripheral blood mononuclear cells [19]. Acute cod liver oil consumption reduced circulating level of TNF*α* and intercellular adhesion molecule-1 (ICAM-1) in healthy individuals [20]. These studies suggest that inhibition of inflammatory cell adhesion/migration induced by inflammatory cytokine TNF*α* may partially explain the protective effects of the fish-oil-rich diet against vascular inflammation.

### 2.2. Dietary Fiber

A high intake of fiber-rich carbohydrates might contribute to weight management and is beneficial for reducing the risk of cardiovascular diseases and diabetes. As a mucilaginous material prepared from the seed husk of plants of the *Plantago* genus, psyllium is an excellent source of mainly soluble fiber. Prolonged feeding of a 3.5% *P. ovata* husk-supplemented diet prevented endothelial dysfunction and the development of hypertension in obese Zucker rats. The diet also decreased body weight gain, reduced hyperinsulinemia and dyslipidemia, restored plasma adiponectin concentration, and decreased circulating TNF*α* concentration [21]. In diabetic women, dietary intake of whole grain, bran, and cereal fibers decreased circulating C-reactive protein (CRP) and TNFR2 concentration [22].

### 2.3. Natural Products

There is a growing body of literature supporting the beneficial effects of natural products on optimal health and disease prevention. As a group of chemical substances found in plants, polyphenols are characterized by the presence of more than one phenol unit or building block per molecule. The health benefits of several specific polyphenols on vascular dysfunction are well established.

#### 2.3.1. EGCG and other Flavonoid Polyphenolics

Flavonoids are most commonly known for their antioxidative and anti-inflammatory activity. Over 5000 naturally occurring flavonoids have been identified from various plants. Epigallocatechin gallate (EGCG), a green tea catechin, is the major component of green tea flavonoids. EGCG significantly attenuated the elevated TNF*α* levels and impaired vasodilator response to acetylcholine induced by low density lipoprotein (LDL) in a rat model [23]. EGCG decreases TNF*α*-induced fractalkine expression by suppressing nuclear factor-kappa B (NF*κ*B) [24] and inhibited TNF*α*-induced monocyte chemoattractant protein-1 (MCP-1) production and the activation of activator protein-1 (AP-1) in vascular endothelial cells via heme oxygenase-1- (HO-1) dependent mechanisms [25, 26]. Apigenin is a flavone that is the aglycone of apiin. Apigenin profoundly reduced monocytes adhesion to human umbilical vein endothelial cells (HUVECs) monolayer by suppressing TNF*α*-stimulated upregulation of vascular cell adhesion protein-1 (VCAM-1), ICAM-1, and E-selectin mRNA expression [27]. As a flavonol, quercetin downregulated TNF*α*-induced ICAM-1 and E-selectin expression in human endothelial cells [28–30]. Isoflavone genistein inhibited TNF*α*-induced apoptosis in human aortic endothelial cells as determined by caspase-3 activation, 7-amino actinomycin D staining, in situ apoptotic cell detection, and DNA laddering [31]. These studies provide a novel mechanism where flavonoids could provide direct vasoprotective benefits in inflammatory cardiovascular diseases by inhibiting TNF*α* and TNF*α*-induced inflammation.

#### 2.3.2. Resveratrol and other Nonflavonoid Polyphenolics

For the last few decades, extensive work has been done to establish the biological activities and pharmacological actions of resveratrol and other nonflavonoid polyphenolics in protecting against vascular dysfunction in various disease states.

Resveratrol is a natural phytophenol that can be extracted from grape skins [32]. Epidemiological studies suggest that Mediterranean diet, which is rich in resveratrol, is associated with reduced risk of cardiovascular diseases [33]. Resveratrol ameliorated endothelial apoptosis by inhibiting TNF*α*-elicited increases in caspase-3/7 activity in endothelial cells and cultured rat aortas [34]. The protective effect of resveratrol was attenuated by the inhibition of glutathione peroxidase and HO-1, suggesting a role for antioxidant systems in the antiapoptotic action of resveratrol [34]. In addition to antiapoptotic effects, resveratrol inhibited TNF*α*-stimulated monocytes adhesion to endothelial cells [35]. The mechanism might be through inhibiting TNF*α*-induced NF*κ*B activation and inflammatory gene expression [36]. Matrix metalloproteinases (MMPs) play an important role in extracellular matrix metabolism, which is a critical contributor to arterial pathology. Resveratrol dose-dependently suppressed TNF*α*-induced proliferation and expression of MMP-9 in vascular smooth muscle cells through the transcription factors NF*κ*B and AP-1 [37]. Upregulated expression of fractalkine is associated with increased atherosclerotic lesion formation in apolipoprotein E^−/−^ (ApoE^−/−^) mice [38]. Resveratrol strongly suppressed TNF*α*-induced fractalkine expression in endothelial cells through the suppression of NF*κ*B and Sp1 transcription factor activities [39]. Resveratrol activates eNOS and increases muscle microvascular blood volume and flow via an NO-dependent mechanism, but systemic infusion of TNF*α* prevented resveratrol-induced muscle microvascular recruitment [40]. Therefore, resveratrol protected against TNF*α*-induced endothelial apoptosis, monocytes adhesion, cell proliferation, atherogenesis, and dysregulation of vasomotor function through multiple signaling pathways. Resveratrol also suppressed plasma TNF*α* concentration following inflammatory stimulation by lipopolysaccharide (LPS) [41] and modulates vascular TNF*α* expression [42]. Aortic TNF*α* expression was significantly increased in type 2 diabetic mice, accompanied by impaired endothelium-dependent vasorelaxation. Chronic resveratrol treatment improved endothelial function and reduced vascular TNF*α* expression [42]. Both anti-TNF*α* treatment and resveratrol markedly inhibited the NF*κ*B pathway through suppressing inhibitor of NF*κ*B (I*κ*B) protein phosphorylation [42, 43], and significantly attenuated vascular oxidative stress and improved NO production. Thus, resveratrol mediated vasculoprotective effects by inhibiting TNF*α* expression and TNF*α*-induced signaling pathway. 

Curcumin is another important natural polyphenol. It inhibited TNF*α*-mediated adhesion of monocytes to endothelial cells by suppression of cell surface expression of adhesion molecules and of NF*κ*B activation [44, 45]. The mechanism was through inhibiting TNF*α*-induced I*κ*B*α* degradation and the nuclear import of NF*κ*B. In contrast, curcumin inhibited AP-1 by direct interaction of curcumin with AP-1 binding to its DNA binding motif, therefore, reduced the expression of endothelial tissue factor (TF), the central mediator of coagulation [46]. Thrombomodulin (TM) functions as a cofactor in the thrombin-induced activation of protein C in the antithrombotic pathway. Curcumin effectively blocked TNF*α*-induced downregulation of TM and endothelial protein C receptor (EPCR) at both mRNA and protein levels in several human endothelial cells [47]. Curcumin also reduced the intracellular reactive oxygen species (ROS) levels, phosphorylation of c-Jun N-terminal kinases (JNK), p38 mitogen-activated protein kinases (p38), and signal transducer and activator of transcription-3 (STAT3), and the expression of ICAM-1, MCP-1, interleukin-8 (IL-8), and lectin-like oxidised LDL receptor-1 (LOX-1) in TNF*α*-stimulated HUVECs [48, 49]. 

In summary, the anti-inflammatory, antithrombotic, antiapoptotic, and antioxidative effects contribute to the health benefits of those nutritional supplements, which protect against vascular damage at least partially by decreasing TNF*α* production and inhibiting TNF*α*-mediated pathological changes through multiple signaling pathways.

## 3. Exercise

There are dichotomies in the effects of exercise on the production of inflammatory cytokines. A cross-sectional study suggested that inflammatory markers, such as CRP, IL-6, and TNF*α*, were lower in older adults with higher levels of exercise and nonexercise activity and in antioxidant supplement users regardless of exercise level [50]. However, a study using treadmill running mice showed that exercise could increase plasma IL-6 concentrations and lung TNF*α* mRNA expression [51]. This dichotomy may be attributed to the intensity of exercise. Chronic heart failure is associated with increased levels of TNF*α* and markers of endothelial damage, including soluble ICAM-1 (sICAM-1) and E-selectin. Whereas acute bouts of exercise lead to an increase in proinflammatory cytokines and markers of endothelial damage; these effects were not seen when exercise was performed chronically in chronic heart failure patients [52]. A 10-week moderate intensity exercise training improved coronary arteriolar endothelial function and reduced TNF*α* level in the myocardial tissue homogenate of type 2 diabetic mice [53]. Interestingly, diet plus exercise training may exert remarkable benefits when either diet or exercise alone could not demonstrate evident benefits. In obese postmenopausal women, diet plus exercise, but not diet alone, decreased plasma levels of CRP, IL-6, sIL-6R, and sTNFR1 and increased basal and postreceptor stimulated lipolysis in both abdominal and gluteal regions [54]. After eccentric exercise in untrained men, Omega-3 fatty acids supplementation attenuates plasma TNF*α* level [55]. These results suggest that diet supplementation in exercise training is effective in reducing chronic inflammation.

## 4. Perspectives

It is of interest to notice that nutrition and exercise have almost always been studied separately although they are closely related and may amplify each other to maximize their beneficial effects. Studies of diet/dietary supplements plus exercise training in animals and human being subjects would be meaningful to explore the mechanisms of dietary supplements and exercise in protecting against vascular dysfunction and the involvement of TNF*α* signaling.

## 5. Concluding Remarks

In conclusion, TNF*α* is known to amplify several signaling pathways leading to vascular inflammation, apoptosis, oxidative stress, and thrombosis, which are key mediators in the pathogenesis of vascular dysfunction. Dietary supplements and/or exercise decrease TNF*α* level and inhibit TNF*α*-mediated pathological changes through multiple signaling pathways, whereby exerting vasoprotective benefits. We believe that further investigations in this exciting field would facilitate the development of dietary supplements and exercise as adjunctive therapies in the management of cardiovascular diseases.

## References

[1] Ding H, Triggle CR (2005). Endothelial cell dysfunction and the vascular complications associated with type 2 diabetes: assessing the health of the endothelium. *Vascular Health and Risk Management*.

[2] Sattar N (2004). Inflammation and endothelial dysfunction: intimate companions in the pathogenesis of vascular disease?. *Clinical Science*.

[3] Zhang H, Park Y, Wu J (2009). Role of TNF-*α* in vascular dysfunction. *Clinical Science*.

[4] Parameswaran N, Patial S (2010). Tumor necrosis factor-*α* signaling in macrophages. *Critical Reviews in Eukaryotic Gene Expression*.

[5] von Fliedner V, Miescher S, Gerain J (1992). Production of tumor necrosis factor-*α* by naive or memory T lymphocytes activated via CD28. *Cellular Immunology*.

[6] Jaffré F, Callebert J, Sarre A (2004). Involvement of the serotonin 5-HT2B receptor in cardiac hypertrophy linked to sympathetic stimulation: control of interleukin-6, interleukin-1*β*, and tumor necrosis factor-*α* cytokine production by ventricular fibroblasts. *Circulation*.

[7] Tumanov AV, Grivennikov SI, Kruglov AA (2010). Cellular source and molecular form of TNF specify its distinct functions in organization of secondary lymphoid organs. *Blood*.

[8] Tartaglia LA, Weber RF, Figari IS, Reynolds C, Palladino MA, Goeddel DV (1991). The two different receptors for tumor necrosis factor mediate distinct cellular responses. *Proceedings of the National Academy of Sciences of the United States of America*.

[9] Woo KS, Chook P, Yu CW (2004). Effects of diet and exercise on obesity-related vascular dysfunction in children. *Circulation*.

[10] Watts K, Beye P, Siafarikas A (2004). Exercise training normalizes vascular dysfunction and improves central adiposity in obese adolescents. *Journal of the American College of Cardiology*.

[11] Stone NJ (1996). Fish consumption, fish oil, lipids, and coronary heart disease. *Circulation*.

[12] Kris-Etherton PM, Harris WS, Appel LJ (2003). Fish consumption, fish oil, omega-3 fatty acids, and cardiovascular disease. *Arteriosclerosis, Thrombosis, and Vascular Biology*.

[13] Rizza S, Tesauro M, Cardillo C (2009). Fish oil supplementation improves endothelial function in normoglycemic offspring of patients with type 2 diabetes. *Atherosclerosis*.

[14] Mayer K, Meyer S, Reinholz-Muhly M (2003). Short-time infusion of fish oil-based lipid emulsions, approved for parenteral nutrition, reduces monocyte proinflammatory cytokine generation and adhesive interaction with endothelium in humans. *Journal of Immunology*.

[15] Goua M, Mulgrew S, Frank J, Rees D, Sneddon AA, Wahle KWJ (2008). Regulation of adhesion molecule expression in human endothelial and smooth muscle cells by omega-3 fatty acids and conjugated linoleic acids: involvement of the transcription factor NF-*κ*B?. *Prostaglandins Leukotrienes and Essential Fatty Acids*.

[16] Wang TM, Chen CJ, Lee TS (2011). Docosahexaenoic acid attenuates VCAM-1 expression and NF-*κ*B activation in TNF-*α*-treated human aortic endothelial cells. *Journal of Nutritional Biochemistry*.

[17] Sanderson P, Calder PC (1998). Dietary fish oil diminishes lymphocyte adhesion to macrophage and endothelial cell monolayers. *Immunology*.

[18] Mayer K, Merfels M, Muhly-Reinholz M (2002). Omega-3 fatty acids suppress monocyte adhesion to human endothelial cells: role of endothelial PAF generation. *American Journal of Physiology*.

[19] James MJ, Gibson RA, Cleland LG (2000). Dietary polyunsaturated fatty acids and inflammatory mediator production. *American Journal of Clinical Nutrition*.

[20] Papageorgiou N, Tousoulis D, Psaltopoulou T (2011). Divergent anti-inflammatory effects of different oil acute consumption on healthy individuals. *European Journal of Clinical Nutrition*.

[21] Galisteo M, Sánchez M, Vera R (2005). A diet supplemented with husks of Plantago ovata reduces the development of endothelial dysfunction, hypertension, and obesity by affecting adiponectin and TNF-*α* in Zucker rats. *Journal of Nutrition*.

[22] Qi L, van Dam RM, Liu S, Franz M, Mantzoros C, Hu FB (2006). Whole-grain, bran, and cereal fiber intakes and markers of systemic inflammation in diabetic women. *Diabetes Care*.

[23] Tang WJ, Hu CP, Chen MF, Deng PY, Li YJ (2006). Epigallocatechin gallate preserves endothelial function by reducing the endogenous nitric oxide synthase inhibitor level. *Canadian Journal of Physiology and Pharmacology*.

[24] Lee AS, Jung YJ, Kim DH (2009). Epigallocatechin-3-O-gallate decreases tumor necrosis factor-*α*- induced fractalkine expression in endothelial cells by suppressing NF-*κ*B. *Cellular Physiology and Biochemistry*.

[25] Zheng Y, Toborek M, Hennig B (2010). Epigallocatechin gallate-mediated protection against tumor necrosis factor-*α*-induced monocyte chemoattractant protein-1 expression is heme oxygenase-1 dependent. *Metabolism*.

[26] Ahn HY, Xu Y, Davidge ST (2008). Epigallocatechin-3-O-gallate inhibits TNF*α*-induced monocyte chemotactic protein-1 production from vascular endothelial cells. *Life Sciences*.

[27] Lee JH, Zhou HY, Cho SY, Kim YS, Lee YS, Jeong CS (2007). Anti-inflammatory mechanisms of apigenin: inhibition of cyclooxygenase-2 expression, adhesion of monocytes to human umbilical vein endothelial cells, and expression of cellular adhesion molecules. *Archives of Pharmacal Research*.

[28] Kobuchi H, Roy S, Sen CK, Nguyen HG, Packer L (1999). Quercetin inhibits inducible ICAM-1 expression in human endothelial cells through the JNK pathway. *American Journal of Physiology*.

[29] Mochizuki M, Kajiya K, Terao J (2004). Effect of quercetin conjugates on vascular permeability and expression of adhesion molecules. *BioFactors*.

[30] Takano-Ishikawa Y, Goto M, Yamaki K (2003). Inhibitory effects of several flavonoids on E-selectin expression on human umbilical vein endothelial cells stimulated by tumor necrosis factor-*α*. *Phytotherapy Research*.

[31] Si H, Liu D (2009). Isoflavone genistein protects human vascular endothelial cells against tumor necrosis factor-*α*-induced apoptosis through the p38*β* mitogen-activated protein kinase. *Apoptosis*.

[32] Bertelli AA, Das DK (2009). Grapes, wines, resveratrol, and heart health. *Journal of Cardiovascular Pharmacology*.

[33] Simopoulos AP (2005). What is so special about the diet of Greece? The scientific evidence. *World Review of Nutrition and Dietetics*.

[34] Ungvari Z, Orosz Z, Rivera A (2007). Resveratrol increases vascular oxidative stress resistance. *American Journal of Physiology*.

[35] Deng YH, Alex D, Huang HQ (2011). Inhibition of TNF-*α*-mediated endothelial cell-monocyte cell adhesion and adhesion molecules expression by the resveratrol derivative, trans-3,5,4′-trimethoxystilbene. *Phytotherapy Research*.

[36] Csiszar A, Smith K, Labinskyy N, Orosz Z, Rivera A, Ungvari Z (2006). Resveratrol attenuates TNF-*α*-induced activation of coronary arterial endothelial cells: role of NF-*κ*B inhibition. *American Journal of Physiology*.

[37] Lee B, Moon SK (2005). Resveratrol inhibits TNF-*α*-induced proliferation and matrix metalloproteinase expression in human vascular smooth muscle cells. *Journal of Nutrition*.

[38] Lesnik P, Haskell CA, Charo IF (2003). Decreased atherosclerosis in CX_3_CR1-/- mice reveals a role for fractalkine in atherogenesis. *Journal of Clinical Investigation*.

[39] Moon SO, Kim W, Sung MJ (2006). Resveratrol suppresses tumor necrosis factor-*α*-induced fractalkine expression in endothelial cells. *Molecular Pharmacology*.

[40] Wang N, Ko SH, Chai W (2011). Resveratrol recruits rat muscle microvasculature via a nitric oxide-dependent mechanism that is blocked by TNF*α*. *American Journal of Physiology*.

[41] Marier JF, Chen K, Prince P, Scott G, del Castillo JRE, Vachon P (2005). Production of ex vivo lipopolysaccharide-induced tumor necrosis factor-*α*, interleukin-1*β*, and interleukin-6 is suppressed by trans-resveratrol in a concentration-dependent manner. *Canadian Journal of Veterinary Research*.

[42] Zhang H, Zhang J, Ungvari Z, Zhang C (2009). Resveratrol improves endothelial function: role of TNF*α* and vascular oxidative stress. *Arteriosclerosis, Thrombosis, and Vascular Biology*.

[43] Zhang H, Park Y, Zhang C (2010). Coronary and aortic endothelial function affected by feedback between adiponectin and tumor necrosis factor *α* in type 2 diabetic mice. *Arteriosclerosis, Thrombosis, and Vascular Biology*.

[44] Kumar A, Dhawan S, Hardegen NJ, Aggarwal BB (1998). Curcumin (diferuloylmethane) inhibition of tumor necrosis factor (TNF)- mediated adhesion of monocytes to endothelial cells by suppression of cell surface expression of adhesion molecules and of nuclear factor-*κ*B activation. *Biochemical Pharmacology*.

[45] Gupta B, Ghosh B (1999). Curcuma longa inhibits TNF-*α* induced expression of adhesion molecules on human umbilical vein endothelial cells. *International Journal of Immunopharmacology*.

[46] Bierhaus A, Zhang Y, Quehenberger P (1997). The dietary pigment curcumin reduces endothelial tissue factor gene expression by inhibiting binding of AP-1 to the DNA and activation of NF-*κ*B. *Thrombosis and Haemostasis*.

[47] Nan B, Lin P, Lumsden AB, Yao Q, Chen C (2005). Effects of TNF-*α* and curcumin on the expression of thrombomodulin and endothelial protein C receptor in human endothelial cells. *Thrombosis Research*.

[48] Kim YS, Ahn Y, Hong MH (2007). Curcumin attenuates inflammatory responses of TNF-*α*-stimulated human endothelial cells. *Journal of Cardiovascular Pharmacology*.

[49] Lee HS, Lee MJ, Kim H (2010). Curcumin inhibits TNF*α*-induced lectin-like oxidised LDL receptor-1 (LOX-1) expression and suppresses the inflammatory response in human umbilical vein endothelial cells (HUVECs) by an antioxidant mechanism. *Journal of Enzyme Inhibition and Medicinal Chemistry*.

[50] Colbert LH, Visser M, Simonsick EM (2004). Physical activity, exercise, and inflammatory markers in older adults: findings from the health, aging and body composition study. *Journal of the American Geriatrics Society*.

[51] Colbert LH, Davis JM, Essig DA, Ghaffar A, Mayer EP (2001). Tissue expression and plasma concentrations of TNF*α*, IL-1*β*, and IL-6 following treadmill exercise in mice. *International Journal of Sports Medicine*.

[52] Niebauer J, Clark AL, Webb-Peploe KM, Coats AJ (2005). Exercise training in chronic heart failure: effects on pro-inflammatory markers. *European Journal of Heart Failure*.

[53] Lee S, Park Y, Zhang C Exercise training prevents coronary endothelial dysfunction in type 2 diabetic mice. *American Journal of Biomedical Sciences*.

[54] You T, Berman DM, Ryan AS, Nicklas BJ (2004). Effects of hypocaloric diet and exercise training on inflammation and adipocyte lipolysis in obese postmenopausal women. *Journal of Clinical Endocrinology and Metabolism*.

[55] Tartibian B, Maleki BH, Abbasi A (2011). Omega-3 fatty acids supplementation attenuates inflammatory markers after eccentric exercise in untrained men. *Clinical Journal of Sport Medicine*.

